# The Relationship Between Daily Activity Level, Posture Distribution, Stepping Patterns, and Cadence in the BCS70 Cohort

**DOI:** 10.3390/s24248135

**Published:** 2024-12-20

**Authors:** Craig Speirs, Matthew Ahmadi, Mark Hamer, Emmanuel Stamatakis, Malcolm Granat

**Affiliations:** 1 PAL Technologies Ltd., Glasgow G4 0TQ, UK; craig.speirs@strath.ac.uk; 2 Department of Computer and Information Sciences, University of Strathclyde, Glasgow G1 1XH, UK; 3 Mackenzie Wearables Research Hub, Charles Perkins Centre, The University of Sydney, Sydney, NSW 2050, Australia; matthew.ahmadi@sydney.edu.au (M.A.); emmanuel.stamatakis@sydney.edu.au (E.S.); 4 School of Health Sciences, Faculty of Medicine and Health, The University of Sydney, Sydney, NSW 2050, Australia; 5 Division of Surgery and Interventional Science, Faculty of Medical Sciences, University College London, London WC1E 6BT, UK; m.hamer@ucl.ac.uk; 6 School of Health and Society, University of Salford, Salford M6 6PU, UK

**Keywords:** accelerometer, activPAL, BCS70, cadence, physical activity, posture, stepping

## Abstract

This study investigated the relationship between stepping-defined daily activity levels, time spent in different postures, and the patterns and intensities of stepping behaviour. Using a thigh-mounted triaxial accelerometer, physical activity data from 3547 participants with seven days of valid data were analysed. We classified days based on step count and quantified posture and stepping behaviour, distinguishing between indoor, community, and recreation stepping. The results indicated significant differences in time spent in upright (2.5 to 8.9 h, *p* < 0.05), lying (8.0 to 9.1 h, *p* < 0.05), and sedentary (7.0 to 13.0 h, *p* < 0.05) postures across activity levels. At higher daily activity levels (10,000–15,000 steps), individuals tended to spend approximately equal time in each posture (8 h lying, 8 h sitting, and 8 h upright). The study found that at lower stepping-defined activity levels, step volumes were driven primarily by indoor stepping, while at higher activity levels, outdoor and recreation stepping were larger contributors. Additionally, stepping classified as indoor had significantly slower cadences compared to outdoor stepping. These findings suggest that the composition and intensity of stepping behaviours vary significantly with daily activity volumes, providing insights that could enhance public health messaging and interventions aimed at promoting physical activity.

## 1. Introduction

In recent years, there has been an increased move from looking at patterns and accumulation of physical behaviour over the waking day to considering behaviour over the full 24 h day [1]. However, while there has been research investigating the relationship between physical activity and specific postures (lying, sitting, and upright) [2,3,4,5,6,7,8], there has been an absence of studies looking at the relationship between daily activity level and the pattern of these activities throughout the entire day.

Typically, research has considered physical behaviour patterns on a per-individual basis, using mean daily times spent in different postures and activities during the observation period [7,9,10] (giving mean time in sedentary/stepping, etc.). Individuals are classified according to these mean values (for example being classified as low active when the average daily step count is between 5000 and 7499) [11]. Relationships between stepping-defined activity levels and patterns of behaviour have been explored [3]. However, individuals may have differing daily levels of physical activity that are driven by a mixture of vocational and leisure activities that can vary during a period of observation. This averaging and classification may mask common patterns of behaviour across individuals when undertaking similar volumes of daily stepping.

There is a growing appreciation that, in addition to the volume of stepping, the duration and intensity of stepping bouts provide significant insights into the impact of stepping activity. An event-based approach allows for the quantification of stepping activity intensity by measuring and quantifying the cadence of each stepping bout [12,13,14]. Previous research has also shown that the composition of stepping activity, including the duration and intensities of stepping bouts may be similar across different individuals on days when they have similar daily activity levels. This relationship between daily stepping activity levels and other postures (upright, sitting, and lying), and the interrelationship between the postures, has not been previously explored. An event-based approach allows for a more detailed analysis of posture by identifying when changes in posture occur and the sequence of and time spent in different postures. By developing a better understanding of the relationship between activity level, posture, and stepping bout composition, we can characterise patterns of behaviour that are associated with higher daily stepping. This would complement existing research demonstrating the health benefits of increased physical activity [15,16] and reduced sedentary behaviour [16,17,18] by supporting the development of more effective public health messaging by identifying modifiable behaviours, such as patterns of sedentary behaviour, where changes are known to be associated with increased physical activity.

This study aims to investigate the relationship between daily activity levels based on step count, time spent in different postures, and the patterns and intensities of stepping bouts.

## 2. Materials and Methods

### 2.1. Study Design

The British Cohort Study 1970 (BCS70) is a longitudinal study following the lives of approximately 17,000 individuals born in England, Scotland, or Wales during a single week in 1970. The study’s age 46 sweep was carried out between 2016 and 2018, with 8581 cohort members participating. A wide range of data were captured in the sweep, including personal, social, and economic data, a range of biomedical measures, and accelerometer-derived physical activity data. In total, 6492 eligible participants consented to wearing the activity monitor.

### 2.2. Physical Activity Measurement

The study used a thigh-mounted triaxial accelerometer (activPAL3 micro; PAL Technologies Ltd., Glasgow, UK) to collect objective physical activity data [19]. The accelerometer was waterproofed and fitted to the midline of the upper thigh’s anterior aspect by a trained nurse during the biomedical assessment. BCS70 cohort members were asked to wear the monitor for seven days, removing the device and returning it by post at the end of the monitoring period. If the device fell off before completing the seven days, participants were asked not to reattach the monitor before returning the device. Participants provided informed consent, and the study received full ethical approval from the NRES Committee South East Coast—Brighton and Sussex.

The activPAL data were downloaded and initially processed using PALbatch version 9.1.0.72 (PAL Technologies Ltd., Glasgow, UK). The data were exported in a format that describes an individual’s physical activity using an event-based approach [19]. Using this approach, each continuous period of a specific type of activity, such as sitting, standing, and taking a stride, is considered a single event. Our analysis used the GHLA algorithm, which uses the accelerometer data to identify a range of activity classes, including sitting, standing, stepping, and lying. Each stride event determined by the algorithm comprises two steps. All adjacent stride events were combined into a single event, termed a stepping event, with the number of steps in this event being twice the number of strides. Stepping events can then be characterised by their duration, the number of steps, and the cadence. Upright containers were defined by combining continuous standing and stepping events, uninterrupted by a sedentary event.

The exported data now quantify time spent at home, which we term the primary locus. Travelling to and from the primary locus tends to involve either using seated transport, cycling, or undertaking a prolonged period of stepping (either to walk to the intended destination or to access public transport), and individuals normally return to the primary locus at night. Using this definition, time in the primary locus is defined as the continuous period surrounding the time in bed period, whose boundaries are events indicating travel to and from the primary locus. These events are defined as follows:

- continuous stepping longer than one minute
- a period of seated transport
- a period of cycling

Initial cleaning of the activity data was carried out using R (version 4.3.3), a programming language used in statistical and data analysis [20]. Participants were included in the analysis if they had at least seven valid days with twenty-four hours of valid physical activity data.

### 2.3. Classification of Days by Step Count

Days were individually classified, based on daily step count and a modification of an established classification of habitual activity levels [11], into seven groups (very inactive: <2500 steps; inactive: 2500–4999 steps; low active: 5000–7499 steps; somewhat active: 7500–9999 steps; active: 10,000–12,500 steps; highly active: 12,500–14,999 steps; very highly active: 15,000 steps+).

### 2.4. Classification of Days by Posture Composition

To investigate the relationship between daily activity levels and time spent in different postures, we assigned a primary posture, lying, sedentary, or upright, to each event based on the GHLA algorithm-determined activity class using the following mapping:

- Lying—primary lying;
- Sedentary—sedentary, secondary lying, or seated transport;
- Upright—quiet standing, stepping, or cycling.

### 2.5. Quantification of Stepping Behaviour by Upright Container

In a previous study, we defined a functional stepping classification heuristic which allows long periods of continuous stepping to be functionally associated with adjacent periods of shorter stepping. This allows us to more accurately differentiate between stepping taking place within a constrained location, such as within the home, which is unlikely to contain long periods of uninterrupted stepping, and stepping in a community or recreation context, where there are opportunities to undertake long periods of continuous stepping, while also allowing for breaks in stepping, as seen in traffic navigation and dog-walking, that occur during periods of outdoor stepping [12]. Using this heuristic, stepping bouts were classified based on the longest period of continuous stepping in the upright container, into three functional groups (indoor stepping: <1 min; community stepping: 1–10 min; recreation stepping: 10 min+). We then further differentiated stepping in upright containers where the longest period of stepping was shorter than one minute to separate stepping occurring within the primary locus from other indoor stepping events. The primary locus was identified as the events occurring between the last transition event of the previous day and the first transition event of the next day. A transition event is an activity indicative of moving between two locations, either continuous stepping longer than 1 min, a period of cycling, or seated transport.

### 2.6. Association of Activity Level with Time Spent in Primary Postures

For each daily activity level, we calculated the distribution of time spent in different postures. A series of one-way ANOVAs were used to test for significant differences in time spent in each primary posture across our activity levels. Using our previously discussed definition of the primary locus, we characterised the distribution of daily time spent within the primary locus for each daily activity level.

### 2.7. Association of Activity Level with Stepping Behaviour

We then explored the relationship between daily activity level and stepping behaviour. All stepping events within an upright container were classified using the longest period of stepping, using a previously described set of thresholds [12]. For each daily activity level, we then calculated the distribution of stepping time across our four categories of stepping behaviour.

Stepping bouts were then further stratified using the cadence of the longest stepping bout in the upright container. We then calculated the distribution of stepping time across all cadences overall, and by stepping behaviour category, for each activity level. We used t-tests with Bonferroni correction to test for sex-specific differences in cadence across stepping behaviour categories for each activity level.

## 3. Results

Of the 5601 individuals for whom accelerometer data were available, 3547 (63%) had 7 days of valid data, giving 24,829 days of physical behaviour to analyse.

### 3.1. Posture Allocation and Activity Level

**Figure 1:**
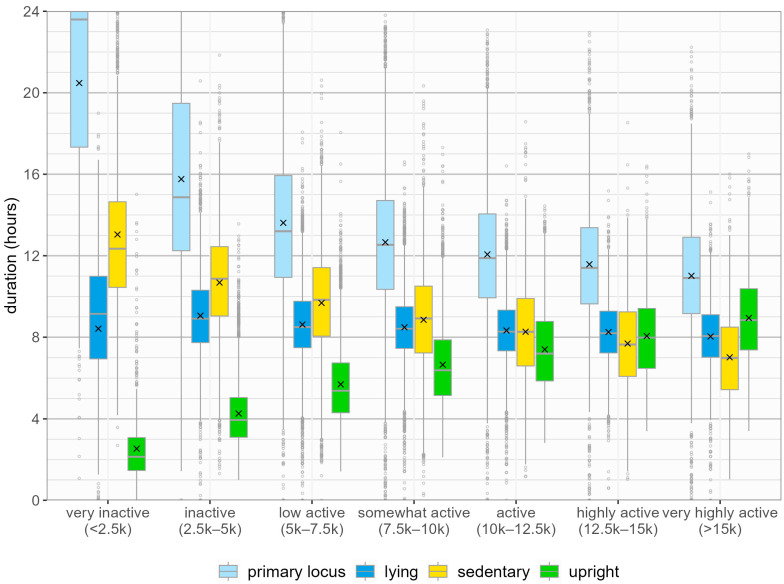
Distribution of daily time in primary postures by activity level. Time attributed to activity spent in the primary locus is also displayed. Data are shown on a daily level, so participants may have data for days at different activity levels.

Figure 1 shows the distribution of daily time spent in each primary posture, stratified by activity level. Significant differences in upright time (F(6,24822) = 2583, *p* < 0.05), lying time (F(6,24822) = 88.93, *p* < 0.05), and sedentary time (F(6,24822) = 1154, *p* < 0.05) were observed between activity levels Compared to days classified as very inactive, very highly active days had more upright time (8.9 h to 2.5 h) and less lying time (8.0 h to 8.7 h) and sedentary time (7.0 h to 12.8 h).

Across the population, most individuals had daily volumes of physical activity that encompassed multiple activity levels (Table 1). Most individuals (54.9%) spent less than half of their observed days in a single activity level, with only a small proportion (0.1%) undertaking activity at the same activity level across the entire observation period.

Individuals undertaking seven days of activity at the same activity level tended to undertake either low (<5000 steps per day) or very high (>15,000 steps per day) daily step volumes (Table 2). When allowing for a single day at a different activity level, individuals were observed to undertake consistent activity at higher daily step volumes (<10,000 steps per day).

### 3.2. Stepping Behaviour and Activity Level

**Figure 2:**
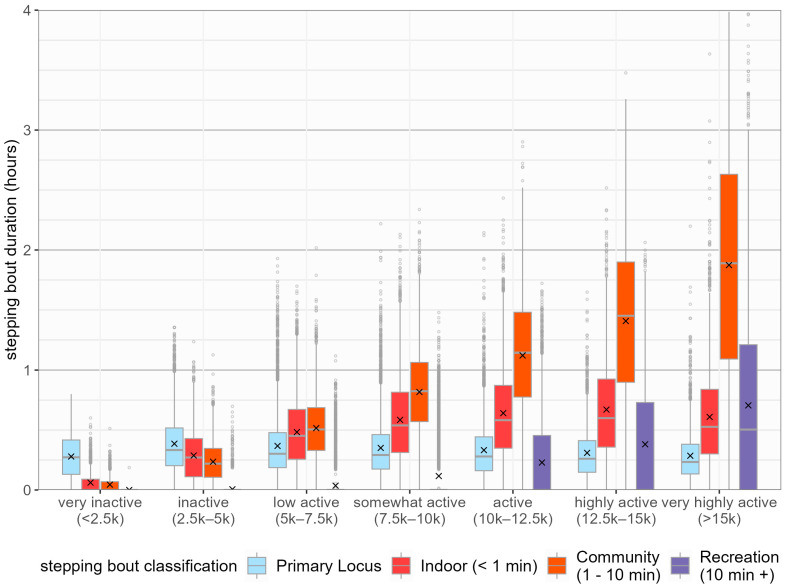
Distribution of daily stepping time by stepping behaviour across activity levels.

Figure 2 shows the distribution of time spent in different stepping behaviours across activity levels. At lower activity levels, increases in step volume were primarily driven by increased volumes of stepping classified as indoor and community. At higher daily stepping volumes, increases in stepping defined as indoor contributed less to increased overall step volume, with recreation stepping only comprising a sizeable proportion of overall stepping at the most intense activity levels.

A breakdown of the number of days within each activity level and the proportion containing recreation stepping is presented in Table 3. As activity level increased, the proportion of days containing recreation stepping increased. There is a near absence of prolonged stepping bouts (longer than 10 min) in days characterised by low activity levels (< 5000 steps per day). The proportion of days containing recreation stepping only exceeded 50% in the highest activity level (Table 4).

When stepping behaviour is simplified to only distinguish between stepping in upright containers with no continuous stepping longer than 1 min (termed indoor) and in upright containers with one or more periods of continuous stepping longer than 1 min (termed outdoor), as shown in Table 3, increases in stepping time defined as indoors stopped at higher activity levels (>7500 steps per day). For stepping classified as outdoors, volumes of stepping consistently increased as activity level increased.

### 3.3. Stepping Cadence and Activity Level

**Figure 3:**
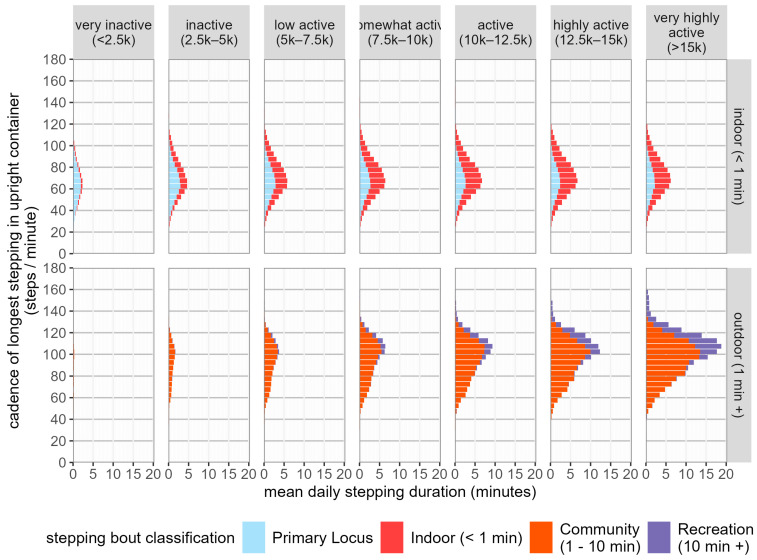
Distribution of stepping time by cadence and stepping behaviour across activity levels.

Figure 3 shows the distribution of stepping cadences at different activity levels. Across all activity levels, the mean cadence of stepping behaviours characterised solely by continuous stepping shorter than 1 min remains broadly stable, while there is a trend to increased cadence of stepping behaviour characterised by the presence of continuous stepping longer than 1 min as activity level increased. Stepping classified as indoor had a distribution of cadences that were significantly slower than the cadence of stepping classified as outdoor.

**Figure 4:**
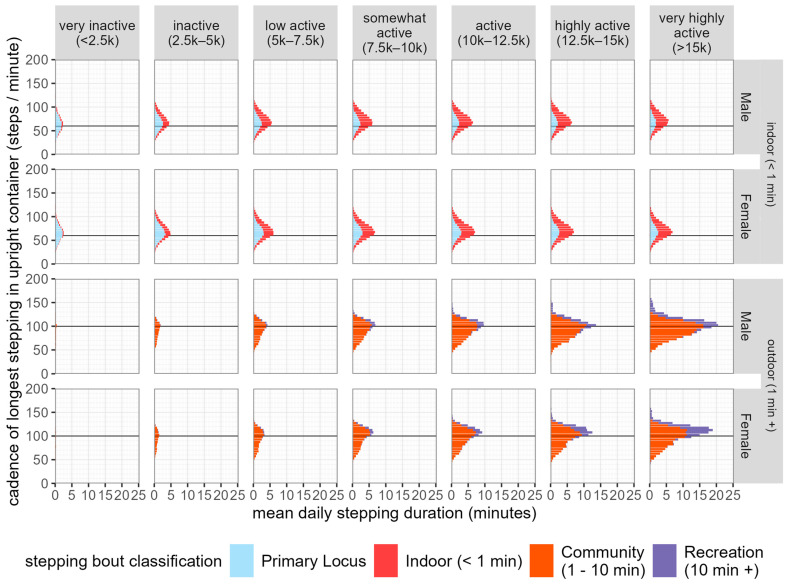
Distribution of stepping time by cadence and stepping behaviour across activity levels. Stepping times are stratified by participant sex.

As shown in Figure 4, between males and females, we observed significant differences (1.24 to 1.72 steps per minute) in the cadence of stepping classified as occurring in the primary locus at most activity levels, except in days characterised by very low (<2500 steps per day) or very high (>15,000 steps per day) activity levels.

In stepping where the longest stepping in upright containers is between 1 and 10 min, termed community stepping, we observed significant differences in cadence between males and females at most activity levels (1.81 to 4.67 steps per minute), except those characterised by low daily step counts (<5000 steps per day). 

## 4. Discussion

This study provides novel insights into how individuals accumulate stepping across stepping behaviours, different postures, and stepping intensities. By using an event-based approach in a cohort of over 3500 UK adults, we were able to characterise 24 h physical behaviour patterns across different activity levels. The observed within-person variation in daily activity level in our population supports our approach of considering each day individually in preference to daily mean values, as used previously [7,9,10].

A key finding was that the way stepping activity is accumulated differs substantially as the activity level increases. At lower daily step counts (<7500 steps), increases were primarily driven by more indoor households and shorter community-based stepping bouts (<10 min). However, at higher activity volumes, longer recreation stepping bouts exceeding 10 min contributed a much larger proportion of the total stepping volume. Across all activity levels, stepping classified as occurring within the primary locus remained relatively constant at approximately 20 min. Given the low proportion of days containing recreation stepping at activity levels corresponding to public health guidelines, this indicates it may not be necessary to undertake prolonged periods of walking to meet public health recommendations for daily stepping [21]. Rather, this stepping can be accumulated through a combination of shorter periods of indoor and community stepping, which is likely associated with daily tasks and transportation needs.

The practical implications of our findings are significant. For inactive individuals not meeting daily stepping guidelines, this study suggests that increasing incidental indoor- and community-based stepping through activities of daily living may be an achievable initial target. As activity levels increase, messaging could then promote incorporating regular bouts of recreational walking to further boost step counts.

The cadence profile of stepping also varied based on activity level and stepping bout context. Indoor stepping cadence profile remained relatively stable regardless of activity volume, likely reflecting the constraints of household ambulation. In contrast, outdoor community and recreation stepping cadence profiles progressively increased at higher activity levels, potentially reflecting greater walking speeds during outdoor travel and exercise [22].

Sex differences in cadence were also observed, particularly for outdoor stepping at moderate-to-high activity volumes. These findings may relate to physiological and socio-cultural factors, including differences in height and the composition of leisure and exercise activities, influencing walking behaviour between males and females [23]. Further investigation may allow further characterisation of these differences and identification of the factors that drive these differences.

The finding that individuals at the level of around 10,000–12,500 steps/day tended to spend approximately equal proportions (~8 h) of the day lying, sedentary, and upright may have practical implications. For example, there may be an optimal balance or composition of physical behaviours that could be targeted through interventions and messaging aimed at achieving moderate-to-high activity levels while avoiding excessive sedentary time. However, further research is needed to determine if this 8:8:8 ratio confers specific health benefits.

While there is compelling evidence demonstrating the health benefits of consistently accumulating appropriate amounts of sleep [24], the health impact of non-sleep-related lying is currently unclear. Future research to quantify the health impact of this behaviour would allow for the development of activity guidelines that account for the differing consequences of these lying behaviours.

This analysis highlights how step count recommendations and walking programmes may need to be tailored based on an individual’s current activity level and lifestyle context. For example, emphasising increasing incidental indoor/community stepping may be most appropriate for inactive individuals, while promoting the incorporation of regular recreational walking could be prioritised for those already achieving higher step counts through occupational or transport-related activities, such as the incorporation of walking breaks for office workers.

A key strength of this study is the use of activPAL, which allowed for precise quantification of postures, stepping behaviours, and walking cadences [25]. However, some limitations should be noted. The study population was a broadly homogenous group of individuals born during a single week in 1970, which, while removing the confounding impact of age, may mean our findings may not be generalisable to other age groups or populations. Additionally, we did not have contextual information about the types of activities people were engaged in, which could further explain differences in stepping patterns. While our exclusion criterion of seven days of valid wear ensures that each participant contributes equally to the analysis, the exclusion of individuals with fewer valid days may introduce bias in our analysis.

## 5. Conclusions

This study highlights that the way stepping activity is accumulated and the balance of different physical behaviours change substantially across the spectrum of daily activity levels. Achieving a sufficient daily step count does not necessarily require regular prolonged bouts of recreational walking. However, at the highest activity levels, recreational stepping comprises a much larger proportion of daily steps. These novel insights can inform the development of tailored physical activity guidance and interventions aimed at optimising the patterns and balance of different physical behaviours for better health. Future research exploring the relationship between patterns of stepping and posture behaviour and long-term health outcomes would support the development of these interventions.

## Figures and Tables

**Table 1:** Breakdown by activity level of the number and proportion of individuals by the maximum number of days spent at a single activity level.

Max Days in Single Activity Level	Individuals	%
7	41	0.1%
6	156	5.2%
5	425	14.0%
4	976	32.2%
3	1431	47.2%
2	518	17.1%
1	0	0%

**Table 2:** Breakdown of the number of days spent at each activity level and the number of individuals who have six or seven days of activity within each activity level. The total number of days reported for each activity level includes days of individuals who spent less than six days within a single activity level.

Daily Activity Level	Step Count	Total Days	Individuals with 7 Days Within Activity Level	Individuals with 6 Days Within Activity Level
very inactive	<2.5 k	869	12	14
inactive	2.5 k–5 k	3685	8	30
low active	5 k–7.5 k	5974	4	46
somewhat active	7.5 k–10 k	5517	1	18
active	10 k–12.5 k	3970	0	5
highly active	12.5 k–15 k	2369	0	1
very highly active	>15 k	2445	16	42

**Table 3:** Breakdown by activity level of time spent in different stepping behaviours (mean and standard deviations) and proportion of stepping characterised as being outdoors.

Daily Activity Level	Step Count	Total Days	Primary Locus Stepping (Minutes)	Indoor Stepping (Minutes)	Outdoor Stepping (Minutes)	Outdoor Stepping (%)
very inactive	<2.5 k	869	16.8 (10.9)	20.5 (11.0)	2.7 (4.3)	11.6%
inactive	2.5 k–5 k	3685	23.2 (14.5)	40.4 (12.6)	14.5 (10.0)	26.4%
low active	5 k–7.5 k	5974	22.0 (15.6)	51.0 (18.7)	33.1 (15.1)	39.4%
somewhat active	7.5 k–10 k	5517	21.1 (15.1)	56.1 (22.8)	56.0 (18.5)	50.0%
active	10 k–12.5 k	3970	20.0 (14.6)	58.4 (24.6)	81.1 (21.3)	58.1%
highly active	12.5 k–15 k	2369	18.6 (13.5)	58.8 (26.4)	107.3 (24.7)	64.6%
very highly active	>15 k	2445	17.1 (13.2)	53.7 (26.7)	163.9 (52.0)	75.3%

**Table 4:** Breakdown by activity level of the number and proportion of days where one or more periods of recreation stepping (10 min +) were undertaken.

Daily Activity Level	Step Count	Days Containing Recreation Stepping	Total Days	% Days with Recreation Stepping
very inactive	<2.5 k	1	869	0.1%
inactive	2.5 k–5 k	69	3685	1.9%
low active	5 k–7.5 k	499	5974	8.4%
somewhat active	7.5 k–10 k	1223	5517	22.2%
active	10 k–12.5 k	1381	3970	34.8%
highly active	12.5 k–15 k	1108	2369	46.8%
very highly active	>15 k	1439	2445	58.9%

## Data Availability

The data that support the findings of this study are available from https://www.ukdataservice.ac.uk/ (accessed on 1 December 2024). Restrictions apply to the availability of these data, which were used under licence for this study.
